# “…he’s going to be facing the same things that he faced prior to being locked up”: perceptions of service needs for substance use disorders

**DOI:** 10.1186/s40352-023-00213-0

**Published:** 2023-03-02

**Authors:** Sara Beeler, Tanya Renn, Carrie Pettus

**Affiliations:** 1 Jane Addams College of Social Work, University of Illinois Chicago, 1040 W Harrison St., 4420 ETMSW MC 309, Chicago, IL 60607 USA; 2 College of Social Work, Institute for Justice Research and Development, Florida State University, 296 Champions Way, University Center, Building C - Suite 2500, Tallahassee, FL 32306-2570 USA; 3 Justice System Partners, South Easton, MA, United States

**Keywords:** Substance use disorders, Social support, Formerly incarcerated, Unmet treatment needs, Help seeking behaviors

## Abstract

**Background:**

High rates of substance use disorders (SUDs) exist among justice-involved populations (i.e., persons incarcerated or recently released). SUD treatment is crucial for justice-involved populations as unmet treatment need increases reincarceration risk and impacts other behavioral health sequalae. A limited understanding of health needs (i.e. health literacy) can be one reason for unmet treatment needs. Social support is critical to seeking SUD treatment and post-incarceration outcomes. However, little is known about how social support partners understand and influence SUD service utilization among formerly incarcerated persons.

**Methods:**

This mixed method, exploratory study utilized data from a larger study comprised of formerly incarcerated men (*n* = 57) and their selected social support partners (*n* = 57) to identify how social support partners understand the service needs of their loved ones recently released from prison who returned to the community with a diagnosis of a SUD. Qualitative data included 87 semi-structured interviews with the social support partners covering post-release experiences with their formerly incarcerated loved one. Univariates were conducted on the quantitative service utilization data and demographics to complement the qualitative data.

**Results:**

Majority of the formerly incarcerated men identified as African American (91%) averaging 29 years of age (SD = 9.58). Most social support partners were a parent (49%). Qualitative analyses revealed that most social support partners avoided using or did not know the language to use regarding the formerly incarcerated person’s SUD. Treatment needs were often attributed to focus on peer influences and spending more time at their residence/housing. Analyses did reveal that when treatment needs were recommended in the interviews, social support partners reported employment and education services to be most needed for the formerly incarcerated person. These findings align with the univariate analysis with their loved ones reporting employment (52%) and education (26%) as their most reported service utilized post-release, compared to only 4% using substance abuse treatment.

**Conclusion:**

Results provide preliminary evidence suggesting social support partners do influence the types of services accessed by formerly incarcerated persons with SUD. The findings of this study emphasize the need for psychoeducation during and after incarceration for individuals with SUDs and their social support partners.

Years of mass incarceration have given way to an era of mass reentry (Chamberlain & Wallace, 2016), with 626,000 individuals being released from state and federal institutions in 2016 (Carson, 2018), but approximately 80% of those will be re-arrested within 9 years following their release (Alper & Durose, 2018). This era of mass reentry from prison to the community is complicated by the many challenging conditions during reentry that can increase the chance for reincarceration, including substance use disorders (SUDs), mental health disorders, and physical health issues (Mallik-Kane & Visher, 2008). Treatment for SUDs is a critical component to success during reentry, but access and utilization may be limited to several reasons including being un/underinsured, not having linkage to care in the community upon release, a lack of appropriate care (i.e. severity of use with level of care), or not perceiving a need for treatment (Osher et al., 2012). By using a sample of formerly incarcerated individuals and their social support partners, we seek to understand a gap in knowledge of how the social support persons of formerly incarcerated individuals understand SUDs and how they help their loved ones prioritize help-seeking behaviors, like service utilization.

## Substance use disorders and related service needs

While incarceration rates have declined since 2009 (Kaeble & Cowhig, 2018), the release rates have not declined with around 600,000 people released from state and federal prisons annually (Carson, 2018). For these individuals who are released, there are an array of challenges faced upon reentry with a common challenge being the high prevalence of SUDs (Begun et al., 2016; McKeganey et al., 2015; Wallace et al., 2016). A review of research over the past 50 years focusing on drug and alcohol use among incarcerated individuals has yielded SUD rates ranging from 10 to 61% for men (Fazel et al., 2017). These rates are further complicated by challenging conditions during reentry including increased risk for reincarceration (Mallik-Kane & Visher, 2008).

The high rate of SUDs for this population highlights the need for robust and accessible behavioral health services for those reentering into the community. Substance use can occur in combination of other behavioral health needs/issues, including mental health and physical health (Chandler et al., 2009; Hamilton & Belenko, 2016; Substance Abuse and Mental Health Services Administration (SAMHSA), 2017). Among criminal justice populations, the incidence of co-occurring disorders is much higher than the general population, with one study’s rates as high as eight in 10 formerly incarcerated men reporting more than one chronic health condition (including mental health and substance abuse; Mallik-Kane & Visher, 2008). Relatedly, albeit dated, incarcerated populations in jails and/or prisons with a mental illness show rates of a co-occurring drug and/or alcohol issue from 59 to 72% (Abram & Teplin,^ 1991^; Ditton, 1999). More recently, the standalone prevalence for the report of a substance use or mental health disorders among individuals with multiple arrests is 52% for SUDs and up to 30% for mental health related concerns (see Substance Abuse and Mental Health Services Administration (SAMHSA), 2017; Jones & Sawyer, 2019).

Moreover, Baillargeon et al.’ (2009) study examined the association of severe mental illness and recidivism and found that inmates diagnosed with psychotic disorders had higher rates of drug possession. They also found former inmates with serious mental illness were more likely to have repeat incarcerations compared to their peers with no serious mental illness. While drug possession does not indicate comorbidity of SUDs, it does indicate the increased prevalence of substance use and mental health issues and its impact on recidivism.

Additionally, research has also illuminated that individuals living with substance use disorders who report a co-occurring mental health issue may experience a progression of symptoms for both or either illness (i.e. anxiety or depression; McHugh, 2015; McHugh & Weiss, 2019). This progression can complicate not only the course of treatment offered (e.g. the ability to effectively address both at the same time) but the outcomes of treatment as well (Padwa et al., 2015; Yule & Kelly, 2019). Furthermore, severe and persistent mental illness, such as schizophrenia or major depression, can impair cognitive and social functioning presenting challenges in treatment and recovery for individuals with substance use disorders (DiClemente et al., 2008). These issues are important factors to consider as they may prevent service utilization or help-seeking behavior.

### Service utilization and help-seeking behavior

Perception of treatment need is associated with service utilization (Hamilton & Belenko, 2016), which is closely connected to health literacy, a person’s understanding and perception of health and associated service needs (Paasche-Orlow & Wolf, 2007). The relationship between perception of treatment need and health literacy can impact an individual’s likelihood of receiving services and can ultimately lead to an increase in help-seeking behavior (Mojtabai et al., 2002). Previous research has shown that health literacy increases a person’s ability to communicate with providers and receive adequate care (Lee et al., 2004). Additionally, Mojtabai et al.’ (2002) study examined perceived treatment need among individuals in the general population diagnosed with either a mood disorder, anxiety or SUD. Only 14% of study participants meeting the criteria for SUD perceived a need for treatment, with less than half of those with a SUD (3%) seeking help from a mental health professional.

In one of the few studies examining health literacy among formerly incarcerated individuals, Hadden et al. (2018) discovered that 60% of their sample had low health literacy which was associated with more emergency department visits and less confidence managing medications. The participants were also more likely to have burdensome chronic health conditions, as well as less education. While this study did not screen persons for or center SUDs, these findings suggest that lower health literacy connects to a lack of understanding or confidence in attending to one’s health conditions. Moreover, it suggests that lower health literacy may decrease the chances that formerly incarcerated persons with health conditions will be seeking and/or utilizing the care and services that are most appropriate. Essentially, an increased understanding or literacy of health illnesses, such as SUDs, could increase the chances that formerly incarcerated persons seeking out, utilizing, and maintaining the treatment they need.

Another important indicator that aligns with health literacy and help seeking behavior is that of motivation to pursue treatment for drug and alcohol use (Rolová et al., 2018). DiClemente et al. (2008) highlight the importance of behavior change in the recovery for individuals living with multiple behavioral disorders, including substance use and mental health. Motivational factors related to modifying or changing the amount of drugs or alcohol consumed, which include a person’s perception of need, beliefs/intentions regarding behavior, and a sense of commitment/responsibility for behavior change, need also consider additional mental health concerns being reported (e.g. post-traumatic stress disorder; DiClemente et al., 2008). For example, among a sample of males seeking community-based alcohol and drug treatment, clients coerced into treatment (legal mandate or encouraged from social support) had statistically higher levels of external motivation, compared to non-coerced clients, however there were no differences observed among treatment engagement (Wolfe et al., 2013). This line of research is critical to help seeking behavior for SUDs as there is currently a dearth of literature on behavioral health literacy. More research is needed to understand this gap of SUDs, health literacy, and help seeking behavior.

### Social support and perceived need for SUDs treatment

The complex service need dynamics for persons coping with SUDs are further compounded by strained relationships and dysfunctional family systems (Lander et al., 2013). Research also highlights the consequences of incarceration that are documented at a macro level, particularly the impact incarceration has on families (Davis et al., 2011; Grosholz et al., 2019). This trickle down effect of harm from the formerly incarcerated person to their social support networks and families has been described as ‘secondary punishment’ (Condry & Minson, 2021) and captures the interdependence of reentry experiences and relationships between the affected person and their support person during the reentry process. Families and other close social support networks provide various forms of support including housing, advice, transportation, and financial support to formerly incarcerated persons (Bakken & Visher, 2018). While research has established the importance of the social support person’s role on incarcerated and formerly incarcerated persons and their success during the reentry period (Pettus-Davis, 2021), how a person’s social support system influences service utilization related to SUDs impacts is not extensively studied or well understood (Edwards et al., 2013; Lee et al., 2004).

Edwards et al., (2013) developed a conceptual model reflecting the ways in which health literacy is “shared and supported” among an individual’s social support network and how it impacts an individual’s behavior and decision-making regarding their health (p. 1189). The four areas where health literacy skills and practices were distributed among participants with chronic health conditions and their social networks included: 1) shared knowledge and understanding, 2) accessing and evaluating information, 3) support with communication, and 4) supporting decision making (Edwards et al., 2013, p. 1187-1189). While Edwards et al., (2013) model research is specific to participants with a *long-term health condition*, this parallels the chronic care model of SUDs as drug and alcohol use disorders are progressive and require ongoing, and at times, specialty care for management of symptoms (McLellan et al., 2013). Additionally, research suggests that social support can help establish a foundation for health literacy, including increasing the use of routine and preventative visits, which is particularly important for poor and marginalized populations (Lee et al., 2004), such as formerly incarcerated individuals.

### The present study

The results of this research collectively illustrate how social support partners can influence their loved one’s understanding or perception of health needs and how they seek and access services, like SUDs. The aim of this current study is to provide preliminary identification of how formerly incarcerated persons and their support persons understand the treatment and service needs of individuals with SUDs and how this understanding impacts their service utilization. The mixed method design facilitates identification of how the formerly incarcerated person and social support partner understand treatment needs with the quantitative and qualitative data working to complement one another, or triangulate, one another (Creswell & Plano Clark, 2017). The following questions guided this research study: 1) How do support persons understand the treatment needs of their loved ones (herein “formerly incarcerated person”) with SUDs who recently released from prison? and 2) What are the most perceived need and utilization of services/treatment among the formerly incarcerated sample with substance use disorders?

## Methodology

### Study overview

The research reported in this study includes secondary quantitative and qualitative collected for a larger social support intervention trial. The trial involved incarcerated men diagnosed with SUDs and their social support partner (*n* = 57 men and 57 social support partners). Upon release from prison, the men and their social support partner participated in a group-based intervention to assist men in developing a positive social support network in the community. Data for the current study was originally collected starting in 2009 through 2010; the data includes quantitative data collected from the formerly incarcerated men pre-and post-release and qualitative data from semi-structured interviews with the social support partners participating in the social support intervention trial.

All study participants represented in the quantitative data were incarcerated males in one of 10 prisons in a southeastern state scheduled to be released within 25-45 days to one large urban county. Eligibility requirements for participation in the trial included: 1) positive screen for a substance use disorder, 2) at least 18 years of age, 3) had a planned release to a large urban county in a southeastern state, 4) ability to speak conversational English, and 5) displayed cognitive understanding of the study requirements for participation. If eligibility was met, participants were enrolled prior to their release from prison. Of the 187 men screened for eligibility, 94 of the men were excluded because of not meeting one or more of the inclusion criteria (*n* = 72) or declined to participate in the study (*n* = 22). Of the 93 men eligible for randomization, 36 men were lost prior to randomization leaving a total of 57 participants. Participants in the current study included all participants who completed the pre-release baseline interview regardless of their randomization assignment from the original trial.

Incarcerated study participants provided contact information for up to four social support partners. The social support partners were then contacted to be screened for study eligibility. Eligibility for the social support partners included: 1) refrain from use of illicit substances, 2) did not drink to intoxication on a weekly basis, 3) no histories of violence towards the study participant, 4) no criminal justice involvement within the past year, 5) at least 18 years of age, 6) spoke conversational English, and 7) displayed cognitive understanding of the study requirements for participation. Social support partners that met eligibility criteria and consented to the study participated in the semi-structured interviews that comprised the qualitative data for this current study. University of [*Full name blinded for peer-review]* Human Subjects Review Boards and the Department of Correction Human Subjects Committee approved study protocol.

### Measures

Qualitative interviews were conducted with social support partners prior to their loved one’s release from prison and 3 months after the loved one’s release from into the community. Quantitative data were collected from incarcerated study participants at baseline (prior to release from incarceration), within a week after release from incarceration, immediately after intervention ended, and 6 months after intervention ended. The quantitative data presented in this study will be for sample descriptive characteristics to validate and support qualitative findings (Creswell & Plano Clark, 2017).

#### Qualitative

Qualitative interviews were conducted only with the social support partners that had 1) been selected from their loved one that was formerly incarcerated and 2) met the aforementioned eligibility criteria and gave consent to participant in the social support intervention trial. Qualitative interview questions for the support persons included: (a) When did you feel like you were effective at providing support?; (b) Has there ever been a time when you felt like you did not know how to be more supportive?; (c) What is the most effective thing you can do to help them stay out of trouble?; (d) Have you ever felt that you needed support from someone else or an organization to provide support to your loved one (i.e. the formerly incarcerated participant)?; (e) What is the most satisfying part about being a support person?; (f) What has been the most challenging part about being a support person?; and (g) Is there a transition plan? Since the social support partner qualitative interviews focused on their understanding of the perception of needs for their formerly incarcerated loved one diagnosed with SUDs and did not directly ask about their understanding of addiction or SUDs, it created an important opportunity to highlight what is “top of mind” regarding perceived needs for supporting persons with histories of SUDs.

#### Quantitative

##### Substance use disorder

Study participants were screened for lifetime histories of SUDs using the Substance Abuse Module of the Comprehensive International Diagnostic Instrument (CIDI-SAM). The CIDI-SAM is an assessment tool that has been used with adult populations for clinical and research purposes to screen for and diagnose SUDs using the Diagnostic and Statistical Manual for Mental Disorders (DSM-IV) diagnostic criteria (Cottler et al., 1989), which was the most recent diagnostic criteria at the time of the original study (*citation blinded for peer-review process*). The CIDI-SAM’s test-retest design produced a kappa-value of .82 indicating strong reliability (Cottler et al., 1989).

##### Demographics

Demographic information was gathered from administrative records on the participant prior to release. Demographic variables included: age, race, marital status, drug of choice, drug of choice that causes the most harm (as indicated on the CIDI-SAM), most serious offense, and the relationship type of the support partner enrolled in the study.

##### Service perception & utilization

This measure was developed for the study and asked participants post-release whether they perceived a need for a particular type of service and whether they received the service. Service questions centered on six domains where formerly incarcerated persons with SUDs may have issues: mental health, substance abuse, medical, employment, education, and general social services.

#### Data analysis

An a priori thematic analysis was conducted on the transcripts utilizing an inductive/deductive co-coding process (Padgett, 2017), allowing for broad to narrow focused coding. Two of the research members coded transcripts with one team member designated to lead the analysis and maintain the audit trail to manage and document the data analysis process, analytic decisions, and rationale for those decisions. The two research members met over the course of four meetings to review coding and reach codebook consensus. Percent of agreeability of the codes were 85% indicating strong validity. Once the codebook was confirmed, three themes stemmed from the participants responses regarding how social support partners understand the treatment/service needs of formerly incarcerated persons with SUDs. Additionally, univariates were used to describe sample characteristics of participants and their social support partner relationships, along with perception of service need and service utilization across the six domains. Time point data was aggregated to determine 1) if the participant perceived a service need post-release (0 = no; 1 = yes) and 2) if the participant received a service post-release (0 = no; 1 = yes). Quantitative data was analyzed using StataSE version 15. The two strands of data were collected independently during the parent study and analyzed independently in this secondary mixed methods study. However, the results of the quantitative and qualitative results interacted during the discussion to substantiate and complement each of the findings (Creswell & Plano Clark, 2017).

## Results

### Sample characteristics

All of the formerly incarcerated participants met criteria for a SUD based on the *Diagnostic Statistical Manual for Mental Disorders* (formerly diagnosed as a substance abuse disorder; 4th ed., text rev.; American Psychiatric Association, 2000) using the CIDI-SAM (Cottler et al., 1989). Most incarcerated study participants (*N* = 57) identified as African American or Black (91%) and were not married at time of arrest (93%). Study participants reported a mean age of 29 (SD = 9.58) years old and the most serious offense of participants were similar to state level trends: property offense (37%), violent offense (28%), drug offense (18%), other offense (10%), and sex (7%). Nearly half of support partners were parents, followed by intimate partners and other loved ones: parent (49%), partner/spouse/girlfriend (19%), friend/mother of child (12%), sibling (11%), extended family member (5%), other (2%), and missing (2%).

### Perceived need and service utilization

Service need and service utilization data were compiled for the 46 participants that provided this information (11 participants did not provide post-release service utilization information). Despite the sample’s inclusion criteria of a SUD diagnosis, over 90% (*n* = 42) of participants did not perceive a need for substance abuse treatment. Of the four that perceived treatment need for their substance use, only two (50%) utilized the services. Low perception of need was also identified for mental health services (15%, *n* = 7) and medical services (26%, *n* = 12). Most participants that perceived need for mental health or medical services utilized the service (86 and 75%, respectively), which is a marked increase compared to substance abuse treatment. Education and general social services were the most perceived service needs post-release followed by employment services (59% v 54%, respectively). In terms of services utilized, employment services were the highest utilized (52%), followed by general social services (41%), and then education (26%). See Table 1 for full demographics and sample characteristics.

**Table 1:** Demographics & post-release service characteristics

	*M/N*	*SD*/%
**Demographics (*N*** **= 57)**		
Age	29.02	9.58
Race		
*White*	5	8.77
*African American*	52	91.23
Marital status		
*Not married*	53	92.98
*Married*	4	7.02
Drug of choice		
*Alcohol*	3	5.26
*Cocaine/crack*	1	1.75
*Marijuana*	10	17.54
*Polysubstance*	13	22.81
*Ecstasy*	14	24.56
*Heroin*	0	0.00
*Other*	6	10.53
*N/A*	10	17.54
Most serious offense		
*Violent*	16	28.07
*Sex*	4	7.02
*Drug*	10	17.54
*Property*	21	36.84
*Other*	6	10.53
Support Partner Relationship		
*Parent*	28	49.12
*Sibling*	6	10.53
*Extended Family*	3	5.26
*Partner/spouse/girlfriend*	11	19.30
*Friend/Mother of their child*	7	12.28
*Other*	1	1.75
*Missing*	1	1.75
**Post-Release (*N*** **= 46)**		
Perceived Service Need	No (%)	Yes (%)
*Medical*	34 (73.91)	12 (26.09)
*Substance Abuse*	42 (91.30)	4 (8.70)
*Mental Health*	39 (84.78)	7 (15.22)
*Employment*	21 (45.65)	25 (54.35)
*Education*	19 (41.30)	27 (58.70)
*General Social Services*	19 (41.30)	27 (58.70)
Service Utilization		
*Medical*	37 (80.43)	9 (19.57)
*Substance Abuse*	44 (95.65)	2 (4.35)
*Mental Health*	40 (86.96)	6 (13.04)
*Employment*	22 (47.83)	24 (52.17)
*Education*	34 (73.91)	12 (26.09)
*General Social Services*	27 (58.70)	19 (41.30)

*M* mean, *SD* standard deviation

### Qualitative themes

Three salient themes surfaced from the qualitative data gathered from: 1) A non-clinical perspective of substance use disorder (SUDs), 2) Post-incarceration challenges, and 3) Emphasis on non-treatment needs.

#### A non-clinical perspective of substance use disorders

Many support persons had difficulty in using, avoided using, or did not know the direct language to use, regarding their loved one’s SUD. In most cases when referring to substance abuse, the non-direct language focused on being *around* drugs and/or alcohol or *selling* drugs (rather than using them), disappearing from their residence, hanging out with bad influences (i.e. peers), or being out in the streets. One support person referred to their loved one’s substance use as a “habit” and explained what happened when he indulged in his habit and said “[w]hen he’d get missing and we didn’t hear from him, we were so scared for him. We would um, wait a while and we’d call downtown to see if he was there and were like (sigh of relief). I know that sound terrible…”.

Another support person stated, “I just need to see that he does not hang around the wrong people because that’s his downfall or if something upsets him you know really bad he’ll go running to that…” In another interview, a support person spoke to the power of peer pressure and communicated that his family members would come over and “talk him into things” and would go out and do things he did not need to be doing. Support persons described these issues almost as a pattern that was influenced by factors in their loved one’s environment. A mother described the conflicting feelings she experienced during her son’s release from prison:

I told him that I feared for him being home as well, you know, because I know that once he comes home he’s going to be facing the same things that he faced prior to being locked up... So being incarcerated was a bad thing, but it was a good thing because he was safe…[.]

Another issue described from support persons was the ‘attitude’ of their loved ones that drove their behaviors. Specifically, this was depicted as their loved one going against their advice, acting compulsively, and anger issues. For instance, one support person described how their loved one went against their better advice saying “if you tell him that he shouldn’t go somewhere, maybe he shouldn’t go out that night or he shouldn’t go, you know, be on out in the street. His mind say ‘go’.” Another support person said their loved one was “doing good about the drugs” but that their loved one’s issue was his anger.

Most of the perceived need by support partners focused on getting away from the negative influences, as opposed to the need for SUD treatment. Moreover, most of what the support persons described is common among people with SUDs, yet none of the social support partners explicitly acknowledged this. This speaks to a lack of perceived need that was consistent with the descriptive characteristics as a majority of the study sample did not perceive a need for SUDs treatment. However, increased health literacy could increase perception of treatment need, and therefore increase treatment utilization where they can get help with cognitive reframe, alleviate and process anger, obtain sober supports, and structure time appropriately.

#### Post-incarceration challenges

While most social support persons struggled to directly describe their loved ones’ substance abuse issues, there were several support persons that directly acknowledge the drug and/or alcohol use of their loved ones and the challenges it presented for them. Thus, for a small subset of study participants, the social support partners acknowledged the connection of SUDs to problematic post-release behaviors.

One mother described her son’s use as a way for him to deal with his emotional and physical discomfort:

Well, my personal feeling is the only thing you can do for a drug addict is to support him and try to help. There’s nothing else you can do, because they want to do drugs, they’re gonna’ do drugs, you know, you can’t stop ‘em so, you know…basically that’s probably why they started was to cope, now with [him] I think a lot of it had to do with his shoulder and all the pain he was in. You know, he looked to the wrong source for help.

Similarly, an intimate support partner conveyed how her loved one’s substance abuse would continue to spiral unless he realized he needed treatment and said “But I don’t think any resource would help [him], cuz [he] has to want help for himself and to me, right now, he just he doesn’t want help.”

Another support partner described that her loved one needed treatment because he never stops getting high and will only stop when he is caught by police. One mother addressed her son’s addiction directly and was explicit in him needing treatment out of medical necessity and said “He gets kind of ill sometimes. You know how it is. He’s an alcoholic and that’s the way they get… [he left when] he wanted something to drink I think.” While there was limited conversation regarding active substance use treatment utilization of the formerly incarcerated person, one mother described multiple failed attempts at getting her son help:

When you go to all these different doctors and you’re trying to get help and you’re getting all these different answers and no one else can help and it’s like where else do you turn? You know you’re going to the physicians and he needs help. So, who is able to really help us? Nobody… they don’t try to get you that help unless you know the right people and that’s not fair. And I feel like, if they know something that’s good for a child, you should deliver it to the next child just because your child wasn’t sick doesn’t mean you can’t help somebody else child. You don’t know what’s going on-help the next person, like depression and stress. He was so aggravated and mad at the whole world. Probably his self as well. He tried to commit suicide… we’ve been out to mental health already. We also set up an appointment. He got two appointments… there’s a lot of appointments we’re making to get him on the right track. So he has the medicine and stuff that he need.

This quote really highlights the treatment burden that can accompany substance abuse and the how the systems of care respond to behavioral health needs. Other quotes in this theme highlight the use of alcohol and/or drugs to deal with physical and/or emotional discomfort, an inability to quit use, and also a need of their loved one to *want* to seek treatment. The quotes outlined in this section show more explicit language and awareness regarding SUDs and the consequences of continued use, including re-arrest, illness, and more frequent drug use. This theme connects more broadly to the importance of understanding SUDs (i.e. health literacy) and its connection to the perceived need for treatment.

#### Emphasis on non-treatment needs

The final theme captures the language social support persons used in describing what was needed for their loved one post-release. However, SUDs treatments were rarely discussed. The most common need reported was employment, followed by education.

A support person concisely stated: “Well that, that comes first. That the way you have the most, to have a job….” This support person said that her years of unconditional emotional support have not helped her loved one “straighten up” so she believed it would take a job to help him “get back on the right track.” An additional support person described how employment would provide something nothing else could and said “…every other [thing] I could probably support him, but when it comes to him… being the man and being a provider...I can’t do anything in that matter to help him.” One mother acknowledged the issue of her son’s alcohol use and expressed how a job would resolve his alcohol problems:

… [R]eally he’ll kill himself if he does, if he keeps doing that… A regular job uh uh, that’s what he needs but he ain’t gonna’ do it… I’d really like for him to get him a job somewhere and you know kind of show that he’s a grown up… if he could work, he’d be so proud of it till it would solve all his problems. I really do think that.

Sometimes, support persons voiced multiple or competing needs of their loved ones reintegrating from prison. Support persons conveyed ambivalence of knowing exactly what their loved one would need between employment and/or education but hoped their emotional support would help them decide. Collectively, data in this theme suggest that many support partners prioritize employment and education over SUD and other behavioral health treatment. Support persons described employment as an essential need that would either a) structure their time and keep them busy or occupied, or b) build confidence and self-esteem. These findings are also further stressed next to the service description data among the sample indicating employment as the most utilized service.

### Summary

Three qualitative themes were identified in the analysis: 1) a non-clinical perspective of SUDs, 2) post-incarceration challenges, and 3) emphasis on non-treatment needs. The three themes are unique when connected to the understanding that individuals meeting diagnostic criteria for SUDs *must* receive treatment and support in order to achieve sobriety and long-term recovery [e.g. remission and recovery; American Society of Addiction Medicine (ASAM), 2019]. Unfortunately, stigma and stereotypes concerning addiction decrease awareness of SUDs as a chronic, relapsing disease and also prevents treatment seeking behaviors (Hammarlund et al., 2018; Substance Abuse and Mental Health Services Administration (SAMHSA), 2014). The findings suggest that despite advances in defining how the medical field and helping professionals understand SUDs there is still more work that can be done to educate consumers and their social support networks on the disease, progression, and necessary treatments for successful reintegration from prison.

## Discussion

The central focus of this study was the limited amount of health literacy among a justice involved population and how social support partners can be engaged as a pivotal role in addressing SUDs among formerly incarcerated persons. To this end our study results indicate three points. First, perceived treatment/service need is associated with treatment/service utilization. Our quantitative examination revealed a trend of perceived need and utilization across the most of the service domains were over 50%, indicating that the majority of individuals who feel a service is needed will utilize it, if available. Existing research supports our finding as one study on perceived need and treatment utilization among populations with mental health and/or SUDs discovered at least 59% of individuals that perceived a need for treatment sough at least some form of professional help (Mojtabai et al., 2002). A more recent study by Hamilton and Belenko (2016) is one of few studies examining perception of service need among formerly incarcerated men and women; their results found that while perception of service need was low, over half of participants that perceived service need for SUDs treatment received treatment which further supports results in this study.

Second, while our sample included formerly incarcerated men with SUDs, only 4% perceived a need for specific SUD treatment. This finding alone may suggest lower health literacy among individuals with SUDs releasing from prison, which is consistent with other studies examining vulnerable populations with chronic health conditions (Hadden et al., 2018; Hamilton & Belenko, 2016). This finding is especially important as SUD treatment is a critical indicator of successful reintegration (Begun et al., 2016; Quanbeck et al., 2005; Woods et al., 2013) and without it formerly incarcerated persons are more open to relapse, rearrest (Ali et al., 2018; Luther et al., 2011;), and death (Binswanger et al., 2007). Further, this finding speaks to the lack of awareness or perception of SUDs as a disease, much like diabetes or heart disease, outside of the medical and academic fields. This low number of perceived need is likely underreported given the level of stigma associated with seeking treatment or help with SUDs or the shame that comes with the label of “addict” (Hammarlund et al., 2018).

Lastly, our study suggests that social support persons have the potential to be a pivotal piece in getting formerly incarcerated persons to access needed services post-release. Study results indicate that while all participants met diagnostic criteria for a SUD, most of the formerly incarcerated participants and their support partners did not describe SUD treatment as a priority need for support. For both the formerly incarcerated men and the social support partners who voiced service needs, the majority focused on employment as the primary need, followed by education. While research highlights the importance of employment during reintegration by decreasing recidivism upon release from prison (Bahr et al., 2010; Berg & Huebner, 2011), it is still critical that individuals with SUDs get additional behavioral health needs met (Bakken & Visher, 2018).

Rarely did support partners refer to their loved ones as having an addiction or SUDs, which could be interpreted a few ways. First, this could suggest an overall lack of awareness of SUD symptoms or even awareness of treatment. Second, the lack of SUDs reference could indicate a denial or secrecy about the substance abuse of their loved one. Similar to the low perception of treatment need among the formerly incarcerated participants, it is possible there is a similar stigma or shame associated with reporting SUD treatment needs for their loved one, and thus, could impact the aforementioned low perception of need observed in the sample.

Third, the social support partner could view the substance use of their loved one as normal behavior, and in turn, not a concern. This would align with the learned behavior and environmental perspectives of SUDs (Guerrini et al., 2014; Margret & Ries, 2016). However, a study examining social networks among incarcerated women with co-occurring depression and SUDs found that, on average, women had a substantial portion of their social network comprised of drinkers and/or drug users, however there was reported high support for SUD treatment and low acceptance of alcohol and drug use across family, friends, and romantic relationships (Nargiso et al., 2014). While this particular study is specific to the social support of formerly incarcerated women, it suggests that even if there is drug and alcohol use and/or SUDs present in social networks, there can still be an awareness or support of SUDs treatment. Finally, this lack of SUDs reference may suggest that SUDs treatment is not the most pressing concern for their loved one’s reintegration, given the social support partner or participant’s perception of other service or treatment needs. There are a myriad of health and service needs prevalent among criminal justice *and* populations diagnosed with SUDs (Cropsey et al., 2012; Mallik-Kane & Visher, 2008). Additionally, populations with co-occurring SUDs can be affected by the treatment burden required of addressing all the treatment or service needs (SUDS treatment v. housing v. employment v. medical care; Kahn et al., 2019). The demands of multiple health needs can create health strain (Agnew, 2006), which may lead to use of drugs or alcohol to temporarily alleviate these competing health demands (Stogner & Gibson, 2011).

## Study results within the context of existing literature – future directions of research and practice

Grosholz and Semenza (2018) found that addressing acute health needs among a predominantly male sample incarcerated in prison found an association between acute care needs and increases in serious behavioral misconduct, yet those with chronic illness were less likely to engage in serious misconduct. The authors suggest that this health strain compounded an already strained environment and could be viewed as “unjust” (Grosholz & Semenza, 2018, p. 1539). While this study is inclusive of the post-release environment, this suggestion translates to this discussion as the reintegration process is often marked as a significant transition that can be marked with stressors and barriers (Pettus-Davis & Kennedy, 2020).

Connecting to the importance of social support, Grosholz et al. (2019) also examined the impact on family health strain on offending among a sample of juveniles; results indicate that vicarious mental health strain of the family member was significantly associated with the juvenile’s subsequent violent offending. In other words, the authors explain the child in the sample may be more likely to “feel” the strain of the family member’s mental health issue (i.e. depression) as opposed to their chronic health issue (i.e. diabetes; Grosholz et al., 2019, p. 17). While the present study is centered on social support broadly and was not specific to adolescent or juvenile populations, results from the qualitative data suggest that social supports were attentive to or “named” the mental health strain of their loved one with a substance use disorder, specifically anger, indicating a potential educational intervention point given the relationships between perception of health need and behavior within the family unit pertaining to mental health.

A study conducted Semenza et al. (2020) found among a longitudinal adult sample (ages 18-25) increase in negative health behaviors were significantly associated with an increased likelihood of future drug use. The study also found that familial mental health issues were significantly associated with an increase in living with a chronical physical health issue as well as increased likelihood of future drug use, while controlling for other health factors (Semenza et al., 2020). Overall, the preliminary results from this mixed method study compliment the extant literature examining the impact of health strain on future drug use and help-seeking behavior. While the findings from this study do not suggest causal inferences, it does support the importance of continued research and understanding health need, health behavior, and social support within the help seeking behavior and recovery of formerly incarcerated individuals with substance use disorders.

The results of this study illuminate unique factors of addiction health literacy, particularly among formerly incarcerated populations, that intersect with the influence of social support partners. A recent study found social support to have significant and positive influence among a sample of Chinese male participants who use drugs and their motivation to abstain from use (Xu et al., 2022). The interconnected nature of interpersonal relationships and their importance in the reentry process (Condry & Minson, 2021; Pettus-Davis, 2021) suggest this has relevance to the social support’s understanding, belief, and value placed on the treatment for substance use disorders and the potential to motivate their loved one to engage in treatment.

The motivation to receive treatment for drug and alcohol use is a crucial dynamic of help seeking behavior (DiClemente et al., 2008). In a case study looking at the post prison experiences for persons who inject drugs, Treloar et al. (2021) center the importance of clients assigning value to needed services to get the intended impact of said service. In other words, a person in need of drug and alcohol treatment could recognize they need the service, but if they do not value the outcome of the service it could have detrimental impacts to their recovery and reentry experience.

Pre-release programming is another opportunity where health literacy can be targeted and enhanced, particularly for substance use disorders, given their economic benefit and resulting recidivism decreases (French et al., 2010). The results of this study show that despite 100% of the sample meeting diagnostic criteria for a SUD only 4% perceived a need for treatment pre-release. Mowen et al. (2019) study examining the differences between pre-release and post-release substance abuse programming (among a sample violent offenders) show that participants receiving pre-release substance abuse treatment programming reported lower levels of drug and alcohol use, compared to individuals who received post-release substance abuse treatment. These results align with other work examining the impact of preparatory intervention programming in treatment to reduce recidivism among persons with sexual violence charges (Renn et al., 2022).

Villman (2021) qualitative study examining desistance and self-regulating strategies among a sample of incarcerated persons in Finland indicates motivation and optimism can be built from multiple factors including environment (programming in prison), timing, and relational (family and social support). Building health literacy programming for substance use disorders shows promise, based on our study results and extant evidence, in helping the social support person and their loved one living with a SUD to cope and understand relationship dynamics, comorbid health needs, and their various treatment options that meet their perceived need(s) and individual values. Collectively, these points highlight the utility of preparing persons living with chronic, acute, and complex social and health needs (such as substance use disorders and involvement with the criminal legal system) for treatment and *motivation* for continued programming (Gideon, 2010).

## Limitations

While there are interesting contributions of this research to the field of social work, public health, and criminal justice, there are limitations to note. First, the smaller sample size prohibits generalization to other criminal justice populations and their service need perception and utilization patterns. Second, while there was co-coding conducted to further strengthen the reliability of results, it is possible there could have been biases from the researchers coding and should be considered when applying results to similar populations. Analysis of socio-cultural influences and linguistics were not within the scope of this work given this area of research are within the early stages, however attention to this is critical in future qualitative methods. Relatedly, social support is broadly defined (i.e. family members, partners, parents, etc.) and includes any support partner identified from the participant recently released from prison; thus, this variable does not account for family dynamics, structure, or relationship type (*Beeler, forthcoming*) which is an important avenue of further research in service utilization and addiction.

Furthermore, the support person was offering their feedback on the struggles experienced and what was most needed by their loved one (i.e. the formerly incarcerated person). While this is an important perspective given the research question, it may not have been the same struggles reported by the reentering loved one. It should also be noted that the participants in the study are representative of one state, so results may not be transferable to the broader formerly incarcerated persons with SUDs and their support networks. Additionally, this study focuses on a formerly incarcerated male sample and results uncovered here may be different for formerly incarcerated women. Lastly, self-report measures utilized in the study can be a limitation as there is no way to guarantee responses were accurate.

## Conclusion

This study, while preliminary and with limitations, offers new factors to the discussion of health literacy programming that are critical to a successful release from incarceration and living with substance use disorders. Our findings suggest there may be a need for intervention development that educates family members on the general criteria for SUDs, the variety of treatment approaches, and how treatment may assist in a transition post-incarceration. Lee et al.’ (2004) research support the impact of low health literacy among marginalized populations and their utilization of healthcare services. Their research suggests that incorporating positive social support into intervention treatment may increase health knowledge, improve overall health conditions, and decrease services such as emergency department visits and other hospitalizations.

Future research should examine the types of relationships (e.g. peers, family) within social support networks and see if there are trends among which types of support (e.g. instrumental, emotional, esteem, tangible) are most effective in transferring health literacy. Findings from this type of research could help inform what, where, and who to place individuals with post-release so that the best reintegration outcomes can be achieved. Future research should also expand on the preliminary and exploratory nature of these findings and consider the intersectional effects of gender, race, and/or ethnicity on perception of treatment need and service utilization. These recommendations are made knowing there also need to be more consistent data collection regarding the additional treatment and service needs of populations diagnosed with SUDs. Specifically, more representation in research is needed across a variety of minoritized and historically excluded identities (including, but not limited to: Black/African American, women, nonbinary, and LGBTQ+ identifying populations); this attention to and focus of the lived experiences with structural and system injustices within the criminal legal and healthcare systems are critical to reforming reentry systems of care (Treloar et al., 2021).

Finally, the results of this research further begs the question: how can pre-release behavioral health interventions, and treatment planning post-release, be enhanced? While drug and alcohol treatment typically treats individuals diagnosed with SUDs, more movement and consistency in including family and loved ones in the treatment process are needed within the pre-release and post-release process. In some treatment settings, family counseling may be an option, however, this is not standard. One enhancement that can be made is starting with education pre- and post-release and including support persons in the process. The education received could review common symptomology present among persons coping with SUDs, common comorbidities experienced with SUDs, along with other related behavioral health needs (i.e. mental health and medical care) that can advance recovery. Including the families of reentering individuals into the critical intervention process speaks to treatment not as an isolated issue, but as a systemic, multidimensional one. These suggestions on intervention programming and research supports the need for transdisciplinary and interprofessional collaboration (i.e. social work, public health, criminal justice, and medical providers) that are necessary for improving outcomes in substance use disorder treatment (Rolová et al., 2021) and reintegration experiences for those releasing from prison into the community (Larsen et al., 2022).

## Data Availability

The data collected and analyzed in this article are not publically available due to privacy and protected information of the study participants. Further information on the parent study can be found here: Pettus-Davis, 2014; Pettus-Davis et al., 2017.
